# Datasets used to discover the microbial signatures of oral dysbiosis, periodontitis and edentulism in humans

**DOI:** 10.1016/j.dib.2016.11.051

**Published:** 2016-11-19

**Authors:** M. Colby Hunter, Alex E. Pozhitkov, Peter A. Noble

**Affiliations:** a Program in Microbiology, Alabama State University, Montgomery, AL 36101, USA; b Department of Oral Health, University of Washington, Box 3574444, Seattle, Washington 98195-7444, USA; c Department of Periodontics, University of Washington, Box 3574444, Seattle, Washington 98195-7444, USA

**Keywords:** Gene Meter, Periodontitis, Edentulism, Caries, Health, 16S rRNA genes, Gene abundances, DNA microarrays

## Abstract

This article provides supporting data for the research article ‘Microbial Signatures of Oral Dysbiosis, Periodontitis and Edentulism Revealed by Gene Meter Methodology’ (M.C. Hunter, A.E. Pozhitkov, P.A. Noble, 2016) [1]. In that article, we determined the microbial abundance signatures for patient with periodontics, edentulism, or health using Gene Meter Technology. Here we provide the data used to make the DNA microarray and the resulting microbial abundance data that was determined using the calibrated probes and the 16S rRNA genes harvested from patients. The first data matrix contains two columns: one is the GenInfo Identifier (GI) numbers of the 16S rRNA gene sequences and the other is the corresponding oral bacterial taxonomy. The probes were then screened for redundancy and if they were found to be unique, they were synthesized onto the surface of the DNA microarrays. The second data matrix consists of the abundances of the 576 16S rRNA genes that was determined using the median value of all individual calibrated probes targeting each gene. The data matrix consists of 16 columns and 576 rows, with the columns representing the 16 patients and the rows representing 576 different oral microorganisms. The third data matrix consists of the abundances of 567 16S rRNA genes determined using the calibrated abundance of all aggregated probes targeting the same 16S rRNA gene. The data matrix of the aggregated probes consists of 16 samples and 567 rows.

**Specifications Table**

**Table:** 

Subject area	*Biology, Microbiology*
More specific subject area	*Periodontics, Edentulism, Caries, Health*
Type of data	*Tables*
How data was acquired	*The GI numbers and corresponding bacterial taxonomy was acquired from the Internet. The relative amounts of 16S rRNA genes were determined using Gene Meter methodology.*
Data format	*Raw and calibrated*
Experimental factors	*No normalization was used. 16S rRNA genes were extracted and amplified from patients with one of the following conditions: periodontitis, edentulism, caries, health. Gene abundances were determined from signal intensities from the DNA microarray using calibrated Gene Meter methodology.*
Experimental features	*Every probe targeting a gene on the microarray was calibrated and those that experimentally fit a Langmuir, Freundlich or linear curve were retained and used for determining gene abundance. Since the equation of each individual probes or aggregated probes is known, we can determine the abundance of a specific gene based upon the calibration of the signal intensity.*
Data source location	*N/A*
Data accessibility	*Data is within this article*

**Value of the data**

• The data matrix could by used by scientists to design their own DNA microarrays.
• Scientists could use the gene abundance data to further examine differences in clinical status among patients with periodontitis, edentulism, caries or health.
• Scientists could use the data to benchmark future studies.

## 1 Data

Table 1 contains the data used to make the tiling DNA microarray. The table consists of two columns and 597 rows; one column contains the GI numbers, and the second column contains the corresponding taxonomic assignment of the 16S rRNA gene sequences.

Tables 2 and 3 contain the abundances of various 16S rRNA genes in each of the patient samples. The abundances in Table 2 are based on the median value of all individual probes targeting a specific 16S rRNA gene, while the abundances in Table 3 is based on the aggregated values of all probes targeting a specific 16S rRNA gene.

Table 2 has 20 columns and 577 rows. The first column contains the GI number, the second and third columns contain the corresponding genus and species name, and the fourth column contains the total number of unique probes targeting the 16S rRNA gene. Columns 5–20 contain the median abundances of the 16S rRNA gene by patient sample determined using individually calibrated probes. Columns 5–8 represent patients with caries. Columns 9–12 represent patients with edentulism. Columns 13–16 represent patients with health. Columns 17–20 represent patients with periodontitis. The second letter in the headings of columns 5–20 indicate if the patient was female (F) or male (M). The number following the second letter is the patient number.

Table 3 has 17 columns and 568 rows. The first column contains the GI number. Columns 2–17 contain the relative abundances of the 16S rRNA gene by patient sample determined using aggregate calibrated probes. Columns 2–5 represent patients with caries. Columns 6–9 represent patients with edentulism. Columns 10–13 represent patients with health. Columns 14–17 represent patients with periodontitis. The second letter in the headings of columns 5–20 indicate if the patient was female (F) or male (M). The number following the second letter is the patient number.

## 2 Experimental design, materials and methods

The study design and sample collection methods have been previously published [2]. That study sequenced amplified 16S rRNA genes obtained from extracting DNA from microbial samples obtained from patients with one of the following conditions: caries, edentulism, periodontitis or health. Another study [1] has analyzed the same amplified rRNA genes using Gene Meter methodology. The Gene Meter methodology was originally described in Refs. [3], [4] and its utility has been demonstrated in Refs. [5], [6].

## Appendix A Supplementary material

Supplementary material Table 1. GI numbers for the 16S rRNA gene sequences of 597 oral bacteria. Table 2. Median abundances for the 576 rRNA genes and 16 patient samples based on individual probe calibration. Table 3. Abundances for the 567 rRNA genes and 16 patient samples based on aggregate probe calibration.
